# Brazilian Multicentre Study on Preterm Birth (EMIP): Prevalence and Factors Associated with Spontaneous Preterm Birth

**DOI:** 10.1371/journal.pone.0109069

**Published:** 2014-10-09

**Authors:** Renato Passini, Jose G. Cecatti, Giuliane J. Lajos, Ricardo P. Tedesco, Marcelo L. Nomura, Tabata Z. Dias, Samira M. Haddad, Patricia M. Rehder, Rodolfo C. Pacagnella, Maria L. Costa, Maria H. Sousa, for the Brazilian Multicentre Study on Preterm Birth study group

**Affiliations:** 1 Department of Obstetrics and Gynaecology, School of Medical Sciences, University of Campinas, Campinas, Brazil; 2 Centre for Studies in Reproductive Health of Campinas (CEMICAMP), Campinas, Brazil; 3 Jundiai School of Medicine, Jundiaí, Brazil; Universidade Federal do Acre (Federal University of Acre), Brazil

## Abstract

**Background:**

Preterm birth rate is increasing and is currently a worldwide concern. The purpose of this study was to estimate the prevalence of preterm birth in a sample of health facilities in Brazil and to identify the main risk factors associated with spontaneous preterm births.

**Methods and Findings:**

This was a multicentre cross sectional study on preterm births in 20 referral obstetric hospitals with a case-control component to identify factors associated with spontaneous preterm birth. Surveillance was implemented at all centres to identify preterm births. For eligible consenting women, data were collected through a post-delivery questionnaire completed with information from all mother-newborn medical records until death or discharge or at a maximum of 60 days post-delivery, whichever came first. The risk of spontaneous preterm birth was estimated with OR and 95%CI for several predictors. A non-conditional logistic regression analysis was then performed to identify independently associated factors. The overall prevalence of preterm birth was 12.3%. Among them, 64.6% were spontaneous and 35.4% therapeutic. In the case-control component, 2,682 spontaneous preterm births were compared to a sample of 1,146 term births. Multivariate analyses identified the following as risk factors for spontaneous preterm birth among women with at least one previous birth: a previous preterm birth (OR_adj_ = 3.19, 2.30–4.43), multiple pregnancy (OR_adj_ = 29.06, 8.43–100.2), cervical insufficiency (OR_adj_ = 2.93, 1.07–8.05), foetal malformation (OR_adj_ = 2.63, 1.43–4.85), polyhydramnios (OR_adj_ = 2.30, 1.17–4.54), vaginal bleeding (OR_adj_ = 2.16, 1.50–3.11), and previous abortion (OR_adj_ = 1.39, 1.08–1.78). High BMI (OR_adj_ = 0.94, 0.91–0.97) and weight gain during gestation (OR_adj_ = 0.92, 0.89–0.95) were found to be protective factors.

**Conclusions:**

The preterm birth rate in these health facilities in Brazil is high and spontaneous preterm births account for two thirds of them. A better understanding of the factors associated with spontaneous preterm birth is of utmost importance for planning effective measures to reduce the burden of its increasing rates.

## Introduction

Preterm birth is a major cause of neonatal morbidity and mortality worldwide [1]. Defined as birth occurring before the 37^th^ week of pregnancy [2], preterm birth can be classified as *spontaneous* (spontaneous onset of labour or following pre-labour premature rupture of membranes - pPROM) or *provider-initiated* (induction of labour or elective caesarean birth for maternal or foetal indications, or other non-medical reasons) [3].

Preterm births are spontaneous in around 75% of the cases, with is a multi-factorial aetiology. The risk factors associated with spontaneous preterm births (SPB) seem to vary by gestational age, and social and environment factors [4]. However, more than 50% of them have no causal factor identified [5]. A previous SPB is the strongest predictor of prematurity [6]. In addition, the occurrence of infections during pregnancy [7]–[9], structural abnormalities of the uterus, especially cervical insufficiency [10], several lifestyle conditions (stress, strenuous work, standing work) and habits (smoking, consumption of alcohol and illicit drugs) [11], young or advanced maternal age, short inter-pregnancy interval and low body mass index [12], and uterine over-distention with multiple pregnancies [13] have been described as increasing the risk of preterm births.

Approximately 25% of preterm births are caused by an intentional interruption of pregnancy. Of those, more than half are related to pre-eclampsia, chronic foetal distress, intrauterine growth restriction, *abruptio placentae*, and placental insufficiency [14].

Preterm birth rates are increasing in almost every country with reliable data.^1^ In the United States, nearly 12% of newborns in 2010 were preterm, and this rate has increased by 30% since 1981 [15]. In Brazil, the official prevalence of preterm births in 2006 was around 6.5%. However, this number was suspected to be underestimated. More recently a population-based data showed a higher prevalence of preterm birth in the country, reaching 10.7% in 2011 [16].

The purposes of the Brazilian Multicentre Study on Preterm Birth (EMIP) [17] were to evaluate the prevalence of preterm births in referral obstetric hospitals, and to identify the main factors associated with SPB in this population.

## Methods

### Ethics Statement

The proposal for this study has been reviewed and approved by the National Council for Ethics in Research and by the Institutional Review Board of each site. Before enrolment, an individual Informed Consent form was signed by each subject after understanding and accepting the study conditions. The confidentiality of identity was ensured regardless of whether the women participated in the study or not. The study totally complies with The Declaration of Helsinki.

The Review Boards of the following institutions reviewed and approved this study: Maternidade Climeério de Oliveira (Salvador, BA), Maternidade Escola Assis Chateaubriand (Fortaleza, CE), Hospital Universitaário da Universidade Federal do Maranhao (Sao Luis, MA), Instituto de Sauúde Elıdio de Almeida (Campina Grande, PB), Hospital Universitaário Lauro Wanderley da Universidade Federal da Paraiba (Joao Pessoa, PB), Instituto de Medicina Integral Prof. Fernando Figueira (Recife, PE), Hospital das Clınicas da Universidade Federal de Pernambuco (Recife, PE), Hospital das Clınicas da Universidade Federal do Paranaá (Curitiba, PR), Instituto Fernandes Figueira (Rio de Janeiro, RJ), Hospital das Clinicas da Universidade Federal do Rio Grande do Sul (Porto Alegre, RS), Faculdade de Medicina de Botucatu da Universidade Estadual Paulista (Botucatu, SP), Hospital da Mulher da Universidade Estadual de Campinas (Campinas, SP), Maternidade Escola de Vila Nova Cachoeirinha (São Paulo, SP), Hospital Estadual de Sumaré (Sumaré, SP), Faculdade de Medicina de Jundiaí (Jundiaı, SP), Hospital das Clınicas da Faculdade de Medicina de Ribeirão Preto da Universidade de Saão Paulo (Ribeirão Preto, SP), Santa Casa de Limeira (Limeira, SP), Santa Casa de São Carlos (São Carlos, SP), Casa Maternal Leonor Mendes de Barros (São Paulo, SP), and Hospital São Paulo da Universidade Federal de São Paulo (São Paulo, SP).

### Study design and location

**Figure 1:**
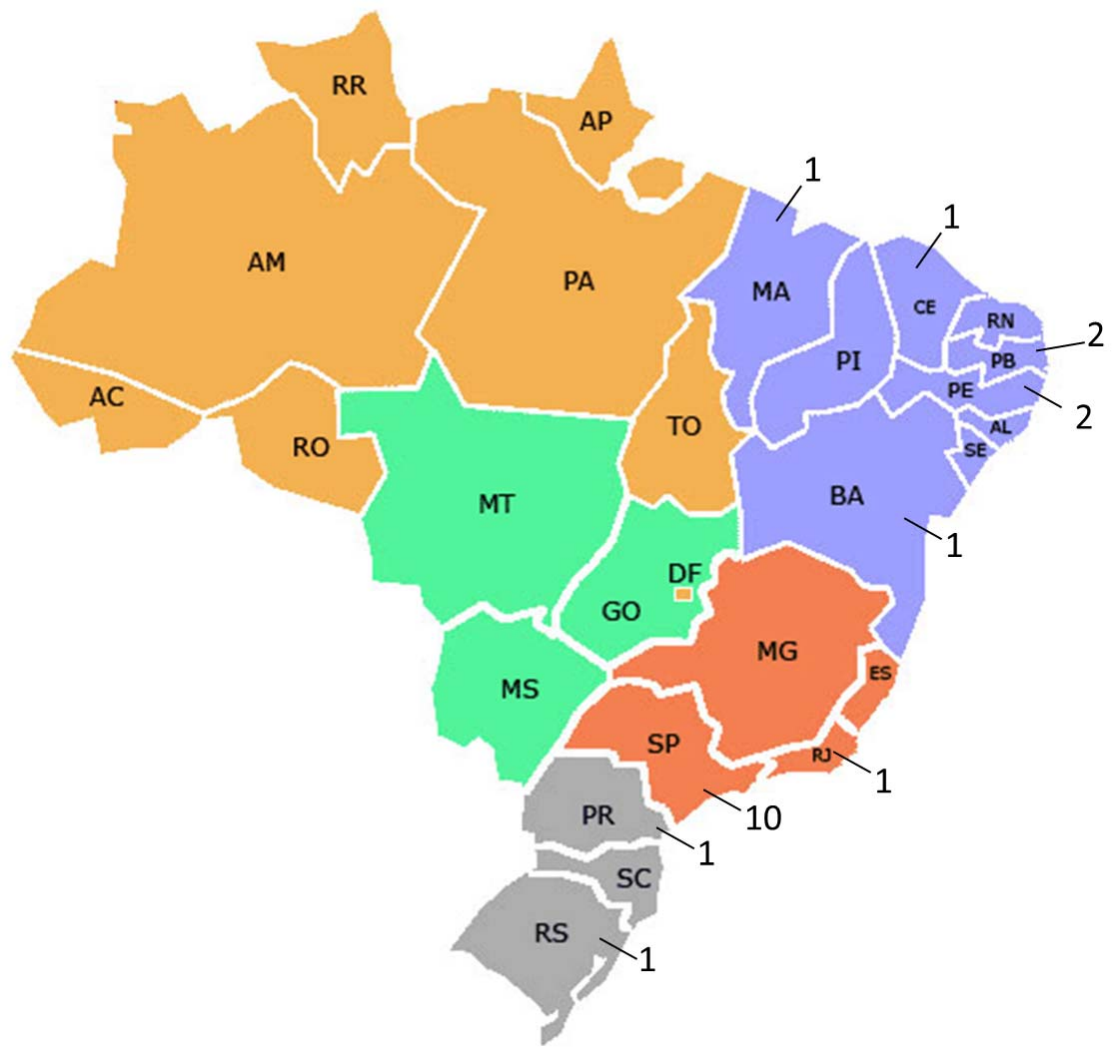
Regional distribution of centres participating in the Brazilian Multicentre Study on Preterm Birth (2 in region South, 7 in region Northeast and 11 in region Southeast).

This is a multicentre cross-sectional study plus a nested case-control component implemented in a research network of 20 referral obstetrical hospitals in different geographical regions of Brazil (Figure 1) [17]. Ranging from a four to nine months period depending on the amount of deliveries, from April 2011 to July 2012, the participating centres performed a prospective surveillance of all patients admitted for delivery, in order to identify preterm births and their main determinant factors. An analysis of risk factors associated with SPB was also planned, comparing women who had preterm birth with a sample of those who delivered at term.

### Sample size

Sample size was calculated using the official prevalence of preterm birth in Brazil in 2006 of 6.5% [16]. Considering an acceptable absolute difference of 0.25% between the sample and the population prevalence, and the probability of type I error of 5%, a sample of 37,000 deliveries would be necessary to screen for preterm births and to have an accurate prevalence estimate of population preterm birth rate [17]. For the sample size related to risk factors, smoking was chosen as associated with preterm birth. Using the estimate of smoking by Brazilian pregnant women of 20% [18], with an OR of 1.4 and the probability of type I error (α) of 0.05 and of type II error (β) of 0.10, 1,054 women would be necessary in each group (“cases” for preterm births and “controls” for term births).

### Study population

This study included women with preterm birth and their newborns admitted during the data collection period, and a random sample of women who delivered at term immediately after the first 1,146 preterm births included (for the case-control component), who agreed to participate in the study. After a preterm birth was identified and enrolled in the study, electronic files and log books of each health facility were checked to identify the term birth that occurred immediately after that specific case of preterm birth. This term birth was then eligible as control if the woman agreed to participate; otherwise the next term birth was approached.

### Variables

The main dependent variable was preterm birth either spontaneous or therapeutic. The independent variables evaluated were related to some socio demographic characteristics, working status, weight assessment, reproductive and obstetrical history, prenatal care (including adequacy of number of prenatal care visits to the gestational age [19]), lifestyle and habits, clinical history, and specific data on short cervix (cervical length below 25 mm between 14 to 24 weeks by vaginal ultrasound scan), cervical insufficiency (any clinical or ultrasound sign), cerclage during pregnancy, uterine fibroid, vaginal bleeding, diagnosis of polyhydramnios, foetal malformation, foetal growth restriction, and multiple pregnancy.

### Data collection procedures

During data collection, each participating centre established a continuous monitoring of preterm births in order to identify women eligible for the study. Once identified, they were invited to participate, received written and verbal explanations, agreed and signed the informed consent, and were enrolled. We approached the term delivery (control) immediately after each preterm birth and the same procedures were followed until the estimated number of controls was achieved.

All information was gathered in a post-delivery interview using a questionnaire designed for the study. Additional relevant information was retrieved from medical records before discharge from the hospital. Data on the newborn was collected at a maximum of sixty days after birth.

A meeting was held with all the participating centres before the start of data collection, in order to standardise the process and procedures for enrolment, data collection and management. Data collection was performed by local researchers who also received an electronic feedback during the study period to remind important points and to address specific questions arising. After completion of each questionnaire, the data were double checked to assess completeness and consistency, and only then introduced in the electronic system database.

### Development of Database

For data entry, a clinical research form (CRF) was developed into the electronic system for the management of clinical studies OpenClinica. Each collaborator received a username and password allowing different types of access to the database depending on their hierarchy in the study. For instance, local researchers had access only to their site information and data entry. Full access was allowed only to those from the coordinating centre. The CRF had an internal consistency checking with a pre-specified range of possible values for each variable in order to avoid data entry errors.

### Data Quality

Several procedures were performed to guarantee high quality and reliable information, including preparatory meetings for training, availability of detailed manuals of interviewer and of operation, technical visits to participating centres, and monitoring of data collection and electronic entry. Auditing and monitoring of collected information were implemented and data changes were provided whenever pertinent after cross-checking.

### Data Analysis

For data analysis we considered a cluster cross-sectional design where each centre corresponded to one cluster. The heterogeneity among clusters was previously checked and considered satisfactory, with very low values of intra-class correlation coefficients for the great majority of variables. Therefore, the reported effect measure was adjusted for the cluster design [20].

The prevalence of preterm birth for the whole sample of the study was estimated as the rate among all births occurring in the participating centres during the data collection period. However not all cases of preterm births were in fact enrolled due to several causes, mainly hospital discharge during weekends before the woman could be approached by research interviewers, and some few cases of refusal to consent. During the study period in the participating facilities there were 4579 preterm births among 37228 births occurred. Considering 4150 preterm births were enrolled (9.37% of eligible women not enrolled), we considered 33740 births to keep the same proportion, also for the regions of the country. Prevalence was then estimated according to the geographical region, gestational age and main determining factor. A bivariate analysis was performed with risk estimates for SPB using OR with 95% confidence intervals (CI) for each predictor.

Then, a multivariate analysis using non-conditional multiple logistic regression was applied to jointly assess the risk factors for SPB, reporting the estimated adjusted odds ratio (OR_adj_) with 95%CI. Two models were run, one including all women and the other only for women with at least one previous pregnancy. The forward selection method was used and only predictors with a p-value <0.10 in the bivariate analysis entered the multivariate model. The software SPSS version 20.0 (SPSS, Chicago, IL, USA), and Stata version 7.0 (StataCorp, College Station, TX, USA) were used for data analysis.

### Role of the funding source

The study was sponsored by two Brazilian governmental agencies which played no other role in the study.

## Results

**Figure 2:**
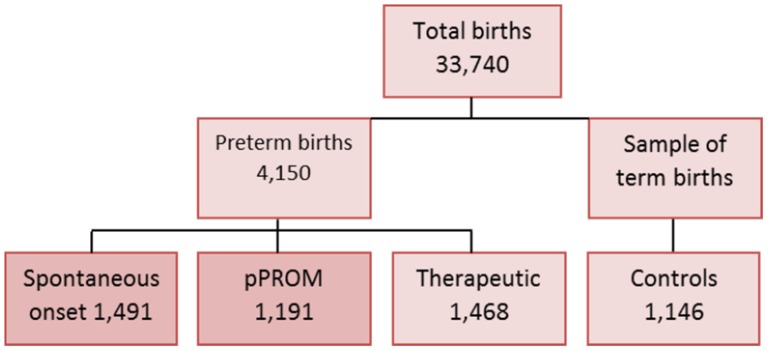
Flow chart of births in the Brazilian Multicentre Study on Preterm Birth.

**Figure 3:**
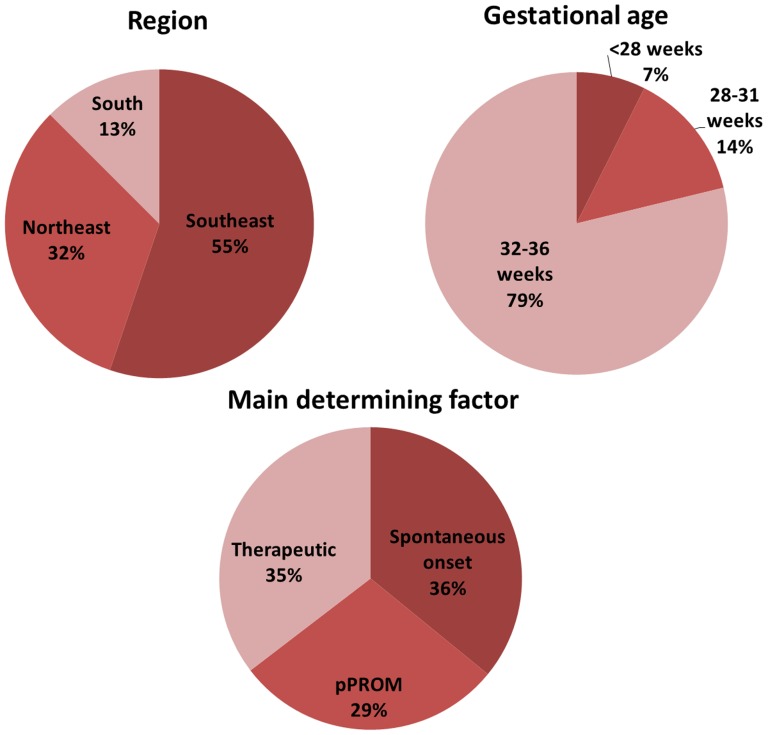
Proportion of preterm births in the Brazilian Multicentre Study on Preterm Birth (EMIP) according to regions, gestational age and main determining factor.

EMIP enrolled a total of 5,296 women, including 4,150 preterm and 1,146 term births. Preterm births included those with spontaneous onset of preterm labour (1,491 cases), pre-labour premature rupture of membranes (1,191 cases), and provider-initiated or therapeutic (1,468 cases), as shown in Figure 2. The total number of births considered for the period of data collection was 33,740 for all facilities (20,565 for the Southeast region, 9,130 for Northeast and 4,045 for South region). The overall prevalence of preterm births was 12.3%, ranging from 14.7% in the Northeast region to 11.1% in the Southeast. Among them, 64.6% were spontaneous and 35.4% therapeutic. Only 7.4% of preterm births occurred below 28 weeks of gestation, while almost 79% were between 32 and 36 weeks (Figure 3, Table 1).

**Table 1:** Prevalence of preterm births in a sample of selected tertiary referral Brazilian maternities according to geographical region, gestational age and main determining factor.

	Preterm births n (%)	Preterm birth rate* (%–95%CI)
BRAZIL	4,150 (100)	12.3 (11.95–12.63)
**Region**		
** Southeast**	2,289 (55.2)	11.1 (10.71–11.57)
** Northeast**	1,341 (32.3)	14.7 (13.97–15.42)
** South**	520 (12.5)	12.8 (11.86–13.92)
**Gestational age**		
** <28 weeks**	308 (7.4)	0.91 (0.81–1.02)
** 28**–**31 weeks**	572 (13.8)	1.70 (1.56–1.84)
** 32**–**36 weeks**	3270 (78.8)	9.69 (9.38–10.01)
**Main determining factor**		
** Spontaneous onset**	1,491 (35.9)	4.42 (4.20–4.64)
** pPROM**	1,191 (28.7)	3.53 (3.34–3.73)
** Therapeutic**	1,468 (35.4)	4.35 (4.14–4.57)

*Total number of births for the period of data collection is 33,740 for all facilities (20,565 for the Southeast region, 9,130 for Northeast and 4,045 for South region).

In the case-control component 2,682 SPB were compared to 1,146 term births. Among the socio-demographic characteristics (Table 2), maternal age ≤19 years (OR 1.54; 1.31–1.79), not having a partner (OR 1.33; 1.08–1.63), and having paid work until the first trimester (OR 2.98; 1.39–6.38) and second trimester (OR 2.43; 1.77–3.35) were significantly associated with SPB. On the other hand, paid work during pregnancy (OR 0.80; 0.65–0.99) and housework (OR 0.59; 0.39–0.90), were negatively associated with SPB.

**Table 2:** Risk estimates for spontaneous preterm birth according to some maternal socio-demographic conditions, comparing women who delivered prematurely (CASES) and women who delivered at term (CONTROLS).

Socio-demographic conditions	CASES	CONTROLS	OR* (95% CI)
	n (%)	n (%)	
**Maternal age (years)**			
** • ≤19**	681 (25.4)	211 (18.4)	**1.54 (1.31**–**1.79)**
** • 20**–**34**	1700 (63.4)	809 (70.6)	Ref.
** • ≥35**	301 (11.2)	126 (11.0)	1.14 (0.84–1.54)
**Skin colour**			
** • White**	1158 (43.2)	451 (39.4)	Ref.
** • Other**	1524 (56.8)	695 (60.6)	0.85 (0.65–1.12)
**Marital status**			
** • With a partner**	2020 (75.3)	919 (80.2)	Ref.
** • Without a partner**	662 (24.7)	227 (19.8)	**1.33 (1.08**–**1.63)**
**Household**			
** • Urban**	2399 (89.9)	1021 (89.5)	Ref.
** • Rural**	269 (10.1)	120 (10.5)	0.95 (0.67–1.35)
**Schooling (years)**			
** • ≤8**	1095 (41.4)	420 (37.2)	1.15 (0.76–1.75)
** • 9**–**12**	1365 (51.6)	629 (55.7)	0.96 (0.65–1.42)
** • >12**	183 (6.9)	81 (7.2)	Ref.
**Family income**			
** • ≥ US$ 400,00**	910 (37.5)	395 (36.4)	Ref.
** • <US$ 400,00**	1519 (62.5)	690 (63.6)	0.96 (0.81–1.13)
**Paid work in pregnancy**			
** • No**	1745 (65.4)	690 (60.3)	Ref.
** • Yes**	923 (34.6)	455 (39.7)	**0.80 (0.65**–**0.99)**
**Paid work until**			
** • First trimester**	68 (7.4)	14 (3.1)	**2.98 (1.39**–**6.38)**
** • Second trimester**	230 (24.9)	58 (12.7)	**2.43 (1.77**–**3.35)**
** • Third trimester**	624 (67.7)	383 (84.2)	Ref.
**Strenuous work**			
** • No**	505 (55.0)	257 (56.6)	Ref.
** • Yes/sometimes**	414 (45.0)	197 (43.4)	1.07 (0.82–1.40)
**Standing work**			
** • No**	355 (38.8)	171 (37.7)	Ref.
** • Yes/sometimes**	561 (61.2)	282 (62.3)	0.96 (0.77–1.19)
**Workload (daily)**			
** • ≤8 hours**	629 (68.9)	324 (71.7)	Ref.
** • >8 hours**	284 (31.1)	128 (28.3)	1.14 (0.78–1.66)
**Night work**			
** • No**	724 (79.6)	355 (78.7)	Ref.
** • Yes**	185 (20.4)	96 (21.3)	0.94 (0.66–1.36)
**Housework**			
** • No**	166 (6.2)	43 (3.8)	Ref.
** • Yes/sometimes**	2515 (93.8)	1102 (96.2)	**0.59 (0.39**–**0.90)**
**Children under 5 years**			
** • No**	1901 (70.9)	821 (71.7)	Ref.
** • Yes**	780 (29.1)	324 (28.3)	1.04 (0.91–1.18)
**Total**	**2,682 (100)**	**1,146 (100)**	

OR*: Odds Ratio adjusted for the cluster effect design; **CI:** confidence interval.

Values in bold mean they are statistically significant.

Table 3 shows that the obstetric history of a previous caesarean section reduced the odds ratio of SPB by around 30% (OR 0.71; 0.61–0.83). On the other hand, all other obstetric conditions evaluated significantly increased the odds ratio of preterm delivery: short inter-pregnancy interval (OR 1.92; 1.38–2.66), previous cerclage (OR 2.35; 1.02–5.40), previous preterm birth (OR 3.05; 2.34–3.98), previous preterm labour (OR 1.79; 1.28–2.50), previous pPROM (OR 1.73; 1.16–2.59) and a previous low birth weight baby (OR 2.78; 2.12–3.65).

**Table 3:** Risk estimates for spontaneous preterm birth according to some maternal obstetric history, comparing women who delivered prematurely (CASES) and women who delivered at term (CONTROLS).

Obstetric history	CASES	CONTROLS	OR* (95% CI)
	n (%)	n (%)	
**Parity**			
** • Nulliparous**	1305 (48.7)	527 (46.0)	0.89 (0.63–1.26)
** • 1**–**2 deliveries**	1021 (38.1)	491 (42.8)	0.75 (0.55–1.03)
** • ≥3 deliveries**	355 (13.2)	128 (11.2)	Ref.
Previous caesarean section^*^			
** • No**	1094 (70.0)	416 (62.4)	Ref.
** • Yes**	469 (30.0)	251 (37.6)	**0.71 (0.61**–**0.83)**
Previous abortion^*^			
** • No**	956 (61.2)	435 (65.2)	Ref.
** • Yes**	607 (38.8)	232 (34.8)	1.19 (0.99–1.43)
Previous uterine curettage^*^			
** • No**	1146 (73.6)	500 (75.2)	Ref.
** • Yes**	411 (26.4)	165 (24.8)	1.09 (0.85–1.38)
Inter-pregnancy interval ^*^			
** • >12 months**	1394 (90.5)	622 (94.8)	Ref.
** • ≤12 months**	146 (9.5)	34 (5.2)	**1.92 (1.38**–**2.66)**
Previous cerclage^*^			
** • No**	1517 (97.6)	656 (98.9)	Ref.
** • Yes**	38 (2.4)	7 (1.1)	**2.35 (1.02**–**5.40)**
Previous preterm birth^*^			
** • No**	1008 (64.7)	565 (84.8)	Ref.
** • Yes**	550 (35.3)	101 (15.2)	**3.05 (2.34**–**3.98)**
Previous preterm labour^*^			
** • No**	1313 (84.5)	604 (90.7)	Ref.
** • Yes**	241 (15.5)	62 (9.3)	**1.79 (1.28**–**2.50)**
Previous pPROM^*^			
** • No**	1312 (84.4)	599 (90.3)	Ref.
** • Yes**	243 (15.6)	64 (9.7)	**1.73 (1.16**–**2.59)**
Previous newborn under 2500g^*^			
** • No**	1090 (71.0)	571 (87.2)	Ref.
** • Yes**	446 (29.0)	84 (12.8)	**2.78 (2.12**–**3.65)**
** • Total**	**2,682 (100)**	**1,146 (100)**	

OR*: Odds Ratio adjusted for the cluster effect design; **CI:** confidence interval; (^*^): excluded Primigravida from the analysis.

Values in bold mean they are statistically significant.

The assessment of other several aspects of pregnancy conditions is shown in Table 4. The absence of prenatal care showed a higher odds ratio of preterm delivery and cases of preterm births were significantly more likely to have prenatal visits performed in a hospital than only in a Primary Health Unit (PHU). In addition, the number of prenatal care visits below that which is expected for an specific gestational age was also significantly associated with preterm births (OR 1.52; 1.19–1.94). The analysis of weight showed that the lower the weight gain during pregnancy, the greater the odds ratio of SPB. Maternal weight gain of up to only 7 kg was more likely, while more than 12 kg of weight gain was less likely to be found among women with SPB. In the same way, higher BMI (≥30) in early or late pregnancy both appeared to decrease the odds ratio of SPB.

**Table 4:** Risk estimates for spontaneous preterm birth according to some conditions during pregnancy, comparing women who delivered prematurely (CASES) and women who delivered at term (CONTROLS).

Conditions during pregnancy	CASES	CONTROLS	OR* (95% CI)
	n (%)	n (%)	
**Prenatal care**			
** • Yes**	2560 (95.5)	1130 (98.6)	Ref.
** • No**	122 (4.5)	16 (1.4)	**3.37 (1.76–6.44)**
**Healthcare facility used for prenatal care**			
** • Only PH**	1585 (61.9)	715 (63.3)	Ref.
** • PHU + hospital**	329 (12.9)	93 (8.2)	**1.60 (1.21**–**2.10)**
** • Only hospital**	444 (17.3)	234 (20.7)	**1.26 (1.10**–**1.45)**
** • Other**	202 (7.9)	88 (7.8)	1.04 (0.66–1.62)
**Initiation of prenatal care**			
** • First trimester**	1395 (64.8)	645 (65.4)	Ref.
** • Second and third trimester**	758 (35.2)	341 (34.6)	1.03 (0.80–1.32)
**Adequacy of number of prenatal care visits**			
** • Adequate**	1539 (67.3)	792 (75.8)	Ref.
** • Inadequate**	749 (32.7)	253 (24.2)	**1.52 (1.19**–**1.94)**
**Weight gain in pregnancy**			
** • ≤7 kg**	839 (36.5)	221 (21.7)	**1.55 (1,23**–**1.95)**
** • 8**–**12 kg**	805 (35.0)	328 (32.3)	Ref.
** • >12 kg**	655 (28.5)	467 (46.0)	**0.57 (0.47**–**0.70)**
**Initial body mass index**			
** • <25 kg/m^2^**	1622(70.4)	655 (64.9)	Ref.
** • 25**–**29.99 kg/m^2^**	442 (19.2)	218 (21.6)	0.82 (0.67–1.00)
** • ≥30 kg/m^2^**	240 (10.4)	137 (13.6)	**0.71 (0.53**–**0.95)**
**Final body mass index**			
** • <25 kg/m^2^**	804(36.4)	197(20.5)	Ref.
** • 25**–**29.99 kg/m^2^**	813 (36.9)	400 (41.5)	**0.50 (0.41**–**0.60)**
** • ≥30 kg/m^2^**	589 (26.7)	366 (38.0)	**0.39 (0.29**–**0.53)**
**Physical effort**			
** • No or rarely**	2149 (80.7)	896 (78.4)	Ref.
** • Yes (often)**	515 (19.3)	247 (21.6)	0.87 (0.69–1.09)
**Depression**			
** • No or rarely**	2296 (86.2)	993 (87.0)	Ref.
** • Yes (often)**	367 (13.8)	149 (13.0)	1.07 (0.80–1.42)
**Anxiety**			
** • No or rarely**	1650 (62.0)	695 (60.8)	Ref.
** • Yes (often)**	1013 (38.0)	448 (39.2)	0.95 (0.76–1.19)
**Use of alcohol**			
** • No**	2217 (83.1)	933 (81.9)	Ref.
** • Yes**	450 (16.9)	206 (18.1)	0.92 (0.64–1.31)
**Smoking (daily)**			
** • No**	2259 (84.2)	1023 (89.3)	Ref.
** • ≤10 cigarettes**	272 (10.1)	84 (7.3)	**1.47 (1.13**–**1.91)**
** • >10 cigarettes**	151 (5.6)	39 (3.4)	**1.75 (1.27**–**2.42)**
**Smoking until (trimester)**			
** • Never or not in pregnancy**	2258 (84.2)	1023 (89.3)	Ref.
** • First and second**	107 (4.0)	27 (2.4)	**1.80 (1.15**–**2.79)**
** • Third**	317 (11.8)	96 (8.4)	**1.50 (1.14**–**1.96)**
**Antenatal substance abuse**			
** • Never**	2522 (94.0)	1105 (96.4)	Ref.
** • Yes or before pregnancy**	160 (6.0)	41 (3.6)	**1.71 (1.15**–**2.55)**
**Vulvovaginitis**			
** • No**	1361 (85.0)	622 (89.1)	Ref.
** • Bacterial vaginosis**	240 (15.0)	76 (10.9)	**1.44 (1.01**–**2.05)**
**Vulvovaginitis**			
** • No**	1389 (86.8)	612 (87.7)	Ref.
** • Candidiasis**	212 (13.2)	86 (12.3)	1.09 (0.76–1.55)
**Urinary tract infection**			
** • No**	1338 (64.5)	645 (70.3)	Ref.
** • Yes**	735 (35.5)	272 (29.7)	**1.30 (1.06**–**1.61)**
**Periodontal infection**			
** • No**	2199 (82.9)	959 (83.9)	Ref.
** • Yes**	455 (17.1)	184 (16.1)	1.08 (0.81–1.44)
**Short cervix (US)**			
** • No**	1047 (95.6)	474 (99.2)	Ref.
** • Yes**	48 (4.4)	4 (0.8)	**5.43 (2.31**–**12.78)**
**Cervical insufficiency (clinical or US)**			
** • No**	2232 (96.4)	976 (99.4)	Ref.
** • Yes**	83 (3.6)	6 (0.6)	**6.05 (2.12**–**17.26)**
**Cerclage**			
** • No**	2361 (97.9)	1003 (99.5)	Ref.
** • Yes**	50 (2.1)	5 (0.5)	**4.25 (1.64**–**10.98)**
**Uterine fibroid**			
** • No**	2308 (98.3)	981 (98.5)	Ref.
** • Yes**	40 (1.7)	15 (1.5)	1.13 (0.64–2.02)
**Vaginal bleeding**			
** • No**	1926 (71.9)	957 (83.6)	Ref.
** • Yes**	751 (28.1)	188 (16.4)	**1.98 (1.60**–**2.46)**
**Anaemia**			
** • No**	1538 (65.6)	750 (72.3)	Ref.
** • Yes**	806 (34.4)	288 (27.7)	**1.36 (1.13**–**1.65)**
**Chronic Hypertension**			
** • No**	2589 (96.6)	1083 (94.6)	Ref.
** • Yes^#^**	92 (3.4)	62 (5.4)	**0.62 (0.45**–**0.86)**
**Chronic Diabetes**			
** • No**	2650 (98.8)	1132 (98.9)	Ref.
** • Yes^#^**	31 (1.2)	13 (1.1)	1.02 (0.50–2.06)
**Gestational hypertension**			
** • No**	2438 (95.4)	1009 (93.5)	Ref.
** • Yes ^#^**	118 (4.6)	70 (6.5)	**0.70 (0.50**–**0.96)**
**Gestational diabetes**			
** • No**	2452 (95.9)	1030 (95.5)	Ref.
** • Yes ^#^**	104 (4.1)	49 (4.5)	0.89 (0.53–1.49)
**Polyhydramnios**			
** • No**	2364 (97.2)	1020 (98.4)	Ref.
** • Yes**	68 (2.8)	17 (1.6)	1.73 (0.84–3.54)
**Foetal malformation**			
** • No**	2333 (94.1)	1043 (98.4)	Ref.
** • Yes**	146 (5.9)	17 (1.6)	**3.84 (2.06**–**7.14)**
Foetal growth restriction^*^			
** • No**	2388 (96.3)	1033 (97.5)	Ref.
** • Yes**	91 (3.7)	27 (2.5)	1.46 (0.68–3.14)
**Multiple pregnancy**			
** • No**	2358 (87.9)	1136 (99.1)	Ref.
** • Yes**	324 (12.1)	10 (0.9)	**15.61 (6.24–39.04)**
**Total**	**2,682 (100)**	**1,146 (100)**	

OR*: Odds Ratio adjusted for the cluster effect design**; CI:** confidence interval**; PHU:** Primary Health Unit.

**(^#^)** Severe and/or complicated cases of maternal hypertension or diabetes that indicated an interruption of pregnancy prematurely were contemplated in therapeutic preterm birth, so excluded from this analysis.

(^*^) Severe cases of foetal growth restriction that indicated an interruption of pregnancy prematurely were contemplated in therapeutic preterm birth, so excluded from this analysis.

Values in bold mean they are statistically significant.

Table 4 also shows that among behavioural characteristics, smoking and antenatal substance abuse were both associated with an increased odds ratio of SPB. Bacterial vaginosis (OR 1.44; 1.01–2.05) and urinary tract infection (OR 1.30; 1.06–1.61) were also identified as risk factors. Some other uterine and pregnancy characteristics were strongly associated with increased odds ratios of SPB, such as short cervix, cervical insufficiency, cerclage during pregnancy, vaginal bleeding during pregnancy, foetal malformation, and multiple pregnancy.

Table 5 shows the results of non-conditional multiple logistic regression analysis with all women whose strongest independent risk factors for SPB identified were multiple pregnancy, followed by foetal malformation, vaginal bleeding, cervical insufficiency, inadequate number of prenatal care visits and urinary tract infection. Higher BMI at the end of pregnancy and weight gain during gestation were both identified as factors associated with a lower proportion of preterm births. For the same analytical approach, including only women with at least one previous pregnancy, Table 6 shows that the factors independently associated with a higher odds ratio of preterm birth were multiple pregnancy, previous preterm birth, vaginal bleeding, foetal malformation, previous abortion, polyhydramnios, and cervical insufficiency. BMI during early pregnancy and weight gain in pregnancy were both again identified as associated with decreased preterm births.

**Table 5:** Variables independently associated with spontaneous preterm birth in all women studied: multiple analyses by non-conditional logistic regression [n = 2,227].

Variables	OR_adj_	95% CI	p-value
Multiple pregnancy	23.56	9.34–59.43	<0.001
Foetal malformation	5.21	3.01–9.03	<0.001
Final body mass index (kg/m^2^)	0.95	0.93–0.97	<0.001
Weight gain in pregnancy (kg)	0.95	0.92–0.97	<0.002
Vaginal bleeding	1.87	1.34–2.61	<0.002
Suspect cervical insufficiency	6.14	1.82–20.71	0.006
Inadequate number of prenatal care visits	1.49	1.12–1.99	0.008
Urinary tract infection	1.28	1.01–1.64	0.044

**OR_adj_:** Odds ratio adjusted for all predictors; **CI:** confidence interval of OR; **p:** p-value.

**Predictors entering the model:** age (years); skin colour (white: 0/other: 1); marital status (with a partner: 0/without a partner: 1); schooling (until 8 years: 1/>8 years: 0); paid work in pregnancy (yes: 1/no: 0); homework (yes, totally or with help: 1/no: 0); parity (until 2: 1/≥3: 0); prenatal care (yes: 0/no: 1); adequacy of number of prenatal care visits (inappropriate: 1/appropriate: 0); weight gain at pregnancy (kg); initial BMI (kg/m^2^); final BMI (kg/m^2^); smoking during pregnancy (no: 0/yes, ≥1 cigarettes: 1); smoking until (0 to 9 months); Antenatal substance abuse (never used: 0/used and stopped at pregnancy, or used at pregnancy: 1); bacterial vaginosis during pregnancy (yes: 1/no: 0); urinary tract infection during pregnancy (yes: 1/no: 0); short cervix (yes: 1/no: 0); cervical insufficiency (yes: 1/no: 0); cerclage (yes: 1/no: 0); vaginal bleeding during pregnancy (yes: 1/no: 0); anaemia during pregnancy (yes: 1/no: 0); change in the volume of amniotic fluid (polyhydramnios: 1/no or oligohydramnios: 0); chronic disease: hypertension (yes: 1/no: 0); gestational hypertension (yes: 1/no: 0); foetal malformation (yes: 1/no: 0); foetal growth restriction (yes: 1/no: 0); other foetal morbidity (yes: 1/no: 0); multiple pregnancy (yes: 1/no: 0).

**Table 6:** Variables independently associated with spontaneous preterm birth in women with at least one previous pregnancy: multiple analyses by non-conditional logistic regression [n = 1540].

Variables	OR_adj_	95% IC	p-value
Previous preterm birth	3.19	2.30–4.43	<0.001
Weight gain in pregnancy (kg)	0.92	0.89–0.95	<0.001
Multiple pregnancy	29.06	8.43–100.2	<0.001
Vaginal bleeding	2.16	1.50–3.11	<0.001
Initial body mass index (kg/m^2^)	0.94	0.91–0.97	<0.001
Foetal malformation	2.63	1.43–4.85	0.004
Previous abortion	1.39	1.08–1.78	0.012
Polyhydramnios	2.30	1.17–4.54	0.019
Suspect cervical insufficiency	2.93	1.07–8.05	0.038

**OR_adj_:** Odds ratio adjusted for all predictors; **CI:** confidence interval of OR.

**Predictors entering the model:** age (years); skin colour (white: 0/other: 1); marital status (with a partner: 0/without a partner: 1); schooling (until 8 years: 1/>8 years: 0); paid work in pregnancy (yes: 1/no: 0); homework (yes, totally or with help: 1/no: 0); parity (until 2: 1/≥3: 0); previous caesarean section (yes: 1/no: 0); previous abortion (yes: 1/no: 0); inter-pregnancy interval (until 12 months: 1/>12 months: 0); previous cerclage (yes: 1/no: 0); previous preterm birth (yes: 1/no: 0); previous preterm labour (yes: 1/no: 0); previous pPROM (yes: 1/no: 0); previous newborn under 2.5 kg (yes: 1/no: 0); prenatal care (yes: 0/no: 1); adequacy of number of prenatal care visits (inappropriate: 1/appropriate: 0); weight gain at pregnancy (kg); initial BMI (kg/m^2^); final BMI (kg/m^2^); anxiety during pregnancy (always:1/rarely or no:0); smoking during pregnancy (no: 0/yes, ≥1 cigarettes: 1); smoking until (0 to 9 months); Antenatal substance abuse (never used: 0/used and stopped at pregnancy, or used at pregnancy: 1); bacterial vaginosis during pregnancy (yes: 1/no: 0); candidiasis during pregnancy (yes: 1/no: 0); urinary tract infection during pregnancy (yes: 1/no: 0); short cervix (yes: 1/no: 0); cervical insufficiency (yes: 1/no: 0); cerclage (yes: 1/no: 0); vaginal bleeding during pregnancy (yes: 1/no: 0); anaemia during pregnancy (yes: 1/no: 0); change in the volume of amniotic fluid (polyhydramnios: 1/no or oligohydramnios: 0); chronic disease: hypertension (yes: 1/no: 0); chronic disease: pulmonary disease (yes: 1/no: 0); gestational hypertension (yes: 1/no: 0); foetal malformation (yes: 1/no: 0); foetal growth restriction (yes: 1/no: 0); other foetal morbidity (yes: 1/no: 0); multiple pregnancy (yes: 1/no: 0).

## Discussion

EMIP represented an innovative and fundamental step of a planned comprehensive assessment of preterm birth in Brazil in order to provide information to support health policies, the implementation of clinical trials, and prevention and treatment strategies. The results showed a higher prevalence of preterm birth than found in other studies. Additionally, this study indicated that multiple pregnancy, previous preterm birth, cervical insufficiency, vaginal bleeding, fetal malformation, polyhydramnios, inadequate prenatal care, previous abortion and urinary tract infection are all factors independently associated with SPB.

The major strengths of EMIP were the expressive number of subjects evaluated and distributed among the three most populous regions of the country and the large number of variables prospectively collected in detail, which allowed for the analysis of several aspects of preterm births. There are, however some limitations in the study that we need to highlight as well. First we were not able to enrol all the eligible women as previously stated. We had a 9.37% rate of loss mainly due to logistic constraints, but we believe this did not represent a selection bias. These losses were similarly distributed among facilities and without a specific pattern of occurrence. In addition, we considered this same rate for having the correspondent number of births in the denominator in order to avoid distortions in the estimates. Additionally some recall bias could also be argued regarding some habits or previous conditions, but we hypothesize that this would be equally distributed between cases and controls. Lastly, the subjects were enrolled mainly from tertiary centres and therefore the results could not be generalized to the whole Brazilian population, but only for those attending centres like the ones from the study for having their deliveries.

The global prevalence rate of preterm birth of 12.3% found in this study was slightly higher than those recently available, ranging from 9.9% to 11.7% [16], [21], [22]. These data confirm the high prevalence of preterm deliveries in Brazil which is one of the highest among countries with similar background. According to the report “Born too Soon” [2]. Brazil stands on the tenth position among the countries with the highest absolute numbers of preterm deliveries. Despite a reduction in mortality rates, the prevalence of preterm birth is increasing in the country, which is in agreement with other studies that describe this trend worldwide, even in high income countries [1]. One possible explanation for this relatively higher rate of preterm birth in the study is that it is not population-based, and data came from tertiary referral obstetric centres, with neonatal intensive care units, which concentrate cases of high risk pregnancies, thus increasing preterm births, especially those which are therapeutically indicated.

Focusing on a large number of predictors, the results of EMIP showed that factors identified as associated with SPB are in accordance with most similar studies. The factor found to have the highest odds ratio was multiple pregnancies, both in parous and nulliparous women. In fact, a previous Brazilian study found an adjusted estimated risk of preterm birth that was almost five times higher among twin pregnancies [21], and a Japanese prospective multicentre study also found multiple pregnancies as a stronger risk factor for preterm birth, besides the short cervical length [23].

Modifications of the uterine cervix and their relation with preterm birth have been largely studied. In EMIP, cervical insufficiency was clearly associated with an increased odds ratio of preterm births, even for first pregnancies. Cervical shortening and the cerclage procedure were associated with a 4- to 6-times higher odds ratio of preterm births in the bivariate analysis. In an international prospective cohort of nulliparous healthy women with a singleton pregnancy, a 4% increased risk of preterm birth was estimated per millimetre decrease in cervical length [24]. When cervical changes are present or insufficiency is suspected, different management strategies have been attempted to prevent preterm birth, including progesterone, cervical cerclage and even cervical pessary [25]–[27].

Prenatal care in Brazil is currently widely available and the number of visits is no longer seen as a real standard of quality. However, one third of women who delivered prematurely had fewer visits than recommended for gestational age [19], and this was associated with a higher odds ratio of preterm birth. Currently, the quality of prenatal care and how adhesion is obtained seems much more important than the number of visits. In fact, some studies showed that the prevention of preterm births is linked to the availability and adequacy of and access to prenatal care that can screen for conditions that may lead to preterm birth [28].

During pregnancy, some conditions such as urinary tract infection and vaginal bleeding were considered risk factors for preterm births; these findings have already been well described in the literature [7]–[9]. In addition, foetal malformation and polyhydramnios were also significantly associated with higher odds ratio of preterm birth, and are generally interconnected. Uterine over-distension increases uterine contractility, but tocolysis in many foetal malformations are not indicated, and therefore polyhydramnios associated with foetal anomalies will eventually lead to preterm delivery. Weight gain during pregnancy and higher body mass index (BMI) values, either early or late in pregnancy, showed a protective effect against preterm delivery, despite the opposite findings of some previous studies on the topic [28], [29]. Studies focusing on risk factors for preterm births found obesity, hypertensive disorders and diabetes mellitus to be positively associated with prematurity [25], [28]; however, they did not separately evaluate spontaneous or therapeutic preterm births, and we believe that their correspondent risk factors are different. The current analysis approached only SPB, then excluding prematurity secondary to maternal and/or foetal diseases determining therapeutic preterm birth. Similar results to those currently presented have already been reported [30]–[31].

There seems to be an interaction between genetic and environmental individual risk factors. The history of a previous SPB was the second strongest condition associated with prematurity in women with at least one previous pregnancy. This is known to be the single most important marker to screen women for in order to select those at higher risk of preterm birth. These findings are in accordance with the literature [6], [28] and support the importance of taking a good history during the first prenatal care visit for an appropriate and timely referral to a special prenatal care unit.

Finally, socio-demographic and behavioural characteristics of women were not significantly associated with SPB. Some other factors such as paid work during pregnancy and housework appeared only in the bivariate analysis as protective. We believe that they are confounded by the common bias that women at higher risk of preterm birth have been removed from these activities and those at lower risk remained working until later in pregnancy. In conclusion, although advances in high-risk obstetric and neonatal care have resulted in the improved survival of infants born prematurely, preterm rates are increasing in Brazil as in other countries. Moreover, this study identified some risk factors for SPB related to pregnancy conditions and maternal care for the Brazilian population that may help to implement health policies. Improving access to and the quality of prenatal care, in order to adequately screen and diagnose conditions and identify risk factors amenable to interventions seem to be worthwhile in order to effectively reduce the burden of preterm birth.
