# Correction: Brazilian Multicentre Study on Preterm Birth (EMIP): Prevalence and Factors Associated with Spontaneous Preterm Birth

**DOI:** 10.1371/journal.pone.0116843

**Published:** 2015-02-06

**Authors:** The PLOS ONE Staff

The following information is missing from the Funding section: Support for this publication was obtained with Grant 2014/1898-7 from the Sao Paulo Research Foundation (FAPESP).
